# Retraction: MiR-182 regulates cell proliferation and apoptosis in laryngeal squamous cell carcinoma by targeting the CRR9

**DOI:** 10.1042/BSR-2019-1348_RET

**Published:** 2022-08-31

**Keywords:** apoptosis, CRR9, laryngeal squamous cell carcinoma, miR-182, proliferation

This article is being retracted from Bioscience Reports at the request of the authors following receipt of a notification from a reader, alerting the Editorial Office to duplicate images that appear across multiple papers. The Hep2/Scramble figure panel in figure 2C shares significant similarities with a figure panel in 5C from the article “MicroRNA-652 suppresses malignant phenotypes in glioblastoma multiforme via FOXK1-mediated AKT/mTOR signaling pathway” (Yang et al 2019) and from figure 4b (after an 180° rotation) from the article “NEAT1/miR-23a-3p/KLF3: a novel regulatory axis in melanoma cancer progression” (Ding et al 2019). The authors wish to retract the article. The Editor-in-Chief and Editorial Board agree with the retraction.

